# A novel *Dictyostelium *RasGEF required for chemotaxis and development

**DOI:** 10.1186/1471-2121-6-43

**Published:** 2005-12-07

**Authors:** Maddalena Arigoni, Enrico Bracco, Daniel F Lusche, Helmut Kae, Gerald Weeks, Salvatore Bozzaro

**Affiliations:** 1 Department of Clinical and Biological Sciences, University of Torino, Regione Gonzole 10, 10043 Orbassano, Italy; 2 Faculty of Biology, University of Konstanz, 78457 Konstanz, Germany; 3 Dept. Microbiology and Immunology, University of British Columbia, Canada V6T1Z3

## Abstract

**Background:**

Ras proteins are guanine-nucleotide-binding enzymes that couple cell surface receptors to intracellular signaling pathways controlling cell proliferation and differentiation, both in lower and higher eukaryotes. They act as molecular switches by cycling between active GTP and inactive GDP-bound states, through the action of two classes of regulatory proteins: a) guanine nucleotide exchange factor (GEFs) and b) GTP-ase activating proteins (GAPs). Genome wide analysis of the lower eukaryote *Dictyostelium discoideum *revealed a surprisingly large number of Ras Guanine Nucleotide Exchange Factors (RasGEFs). RasGEFs promote the activation of Ras proteins by catalyzing the exchange of GDP for GTP, thus conferring to RasGEFs the role of main activator of Ras proteins. Up to date only four RasGEFs, which are all non-redundant either for growth or development, have been characterized in *Dictyostelium*. We report here the identification and characterization of a fifth non-redundant GEF, RasGEFM.

**Results:**

RasGEFM is a multi-domain protein containing six poly-proline stretches, a DEP, RasGEFN and RasGEF catalytic domain. The *rasGEFM *gene is differentially expressed during growth and development. Inactivation of the gene results in cells that form small, flat aggregates and fail to develop further. Expression of genes required for aggregation is delayed. Chemotaxis towards cAMP is impaired in the mutant, due to inability to inhibit lateral pseudopods. Endogenous cAMP accumulates during early development to a much lower extent than in wild type cells. Adenylyl cyclase activation in response to cAMP pulses is strongly reduced, by contrast guanylyl cyclase is stimulated to higher levels than in the wild type. The actin polymerization response to cAMP is also altered in the mutant. Cyclic AMP pulsing for several hours partially rescues the mutant. *In vitro *experiments suggest that RasGEFM acts downstream of the cAMP receptor but upstream of the G protein.

**Conclusion:**

The data indicate that RasGEFM is involved in the establishment of the cAMP relay system. We propose that RasGEFM is a component of a Ras regulated pathway, which integrate signals acting as positive regulator for adenylyl cyclase and negative regulator for guanylyl cyclase. Altered guanylyl cyclase, combined with defective regulation of actin polymerization, results in altered chemotaxis.

## Background

The *ras *proto-oncogenes encode membrane-bound small monomeric GTP-binding proteins with molecular masses ranging between 20 to 40 kDa, which are highly conserved in the course of eukaryotic evolution. Ras proteins control fundamental cell processes including proliferation, differentiation, motility and polarity [1-3]. Like heterotrimeric G proteins they act as molecular switches cycling between two interconvertible forms: inactive, when bound to guanosine diphosphate (GDP), and active, when bound to guanosine triphosphate (GTP) [4]. Conversion between GTP and GDP-bound states is tightly regulated by two set of proteins: guanosine-nucleotide exchange factors (GEFs), and GTPase activating proteins (GAPs). GEF proteins cause activation by catalysing the exchange of bound GDP with GTP, whereas GAPs inactivate Ras by increasing their rate of GTP hydrolysis [5].

Systems composed by GTPases, GAPs and GEFs allow great versatility in the construction of signaling pathways. Signals can be amplified (one GEF could activate several GTPases), integrated (several pathways activate the same GEF and GAP, and the behaviour of one GTPase depends on the total effect of all its GEFs and GAPs), or split (one GTPase induces many effects) [6]. This versatility allows small GTPases to mediate a wide range of different biological functions among different organisms. For example in *S. cerevisiae *RasGEF CDC25 is required for Ras mediated activation of adenylyl cyclase and it is essential for proliferation and spore germination [7], whereas in *Drosophila*, the RasGEF Son-of-sevenless (Sos) functions upstream of Ras in R7 photoreceptor differentiation [8].

Despite its relatively small genome, *Dictyostelium *possesses a relatively large number of *ras *and *rasGEF *encoding genes. *Dictyostelium *is a lower eukaryote with a simple life cycle, consisting of growth and multicellular development, the latter being fully completed in approximately 24 hours. The amoebae live as single cells, growing by feeding on bacteria, which are taken up by phagocytosis, and dividing by binary fission. Upon starvation, cells start releasing the chemoattractant cAMP and gather by chemotaxis to form multicellular aggregates. Within each aggregate, cells differentiate into prespore and several classes of prestalk cells, while undergoing a series of morphogenetic changes, which end up in the formation of fruiting bodies. Fruiting bodies consist of slender stalks of vacuolated, dead cells, bearing on top spores encapsulated in sori.

Here we report the identification and characterization of a novel RasGEF, named RasGEFM. To date only four *Dictyostelium *RasGEFs have been characterized, namely *rasGEFA*, formerly known as *aimless *[9], *rasGEFB *[10], *gbpC *and *gbpD *[11]. The latter two encode for unconventional RasGEFs. GbpC possesses a RasGEF domain coupled to a cyclic nucleotide-binding domain, which is associated with a MAPKKK-like kinase domain, Leucine Rich Repeats (LRR) and a Ras domain. GbpD is highly similar to GbpC, but it lacks the Ras, MAPKKK-like and the LRR domains [11]. These two proteins control myosin phosphorylation and, as consequence, cell motility and chemotaxis [12]. The GEF protein encoded by the *rasGEFA *gene is essential for cell aggregation, acting at the level of adenylyl cyclase activation [9]. *Dictyostelium *cells lacking *rasGEFB *are defective in early development, although they eventually form tiny but normally proportioned fruiting bodies. Furthermore, these cells move unusually rapidly and show severe impairment in cell growth [10].

Here we present evidences that RasGEFM is involved in a Ras regulatory network, required for cAMP receptor dependent signal transduction. Mutant cells lacking the *rasGEFM *gene produce very small, flat aggregates and fail to develop further. Chemotaxis is altered in the mutant, due to inability of the cells to polarize properly. The phenotype can be partially rescued by pulsing cells with cAMP. We show that RasGEFM is involved in controlling cAMP relay and cell motility.

## Results

### Identification, cloning and sequence analysis of *D.d.rasGEFM*

**Figure 1:**
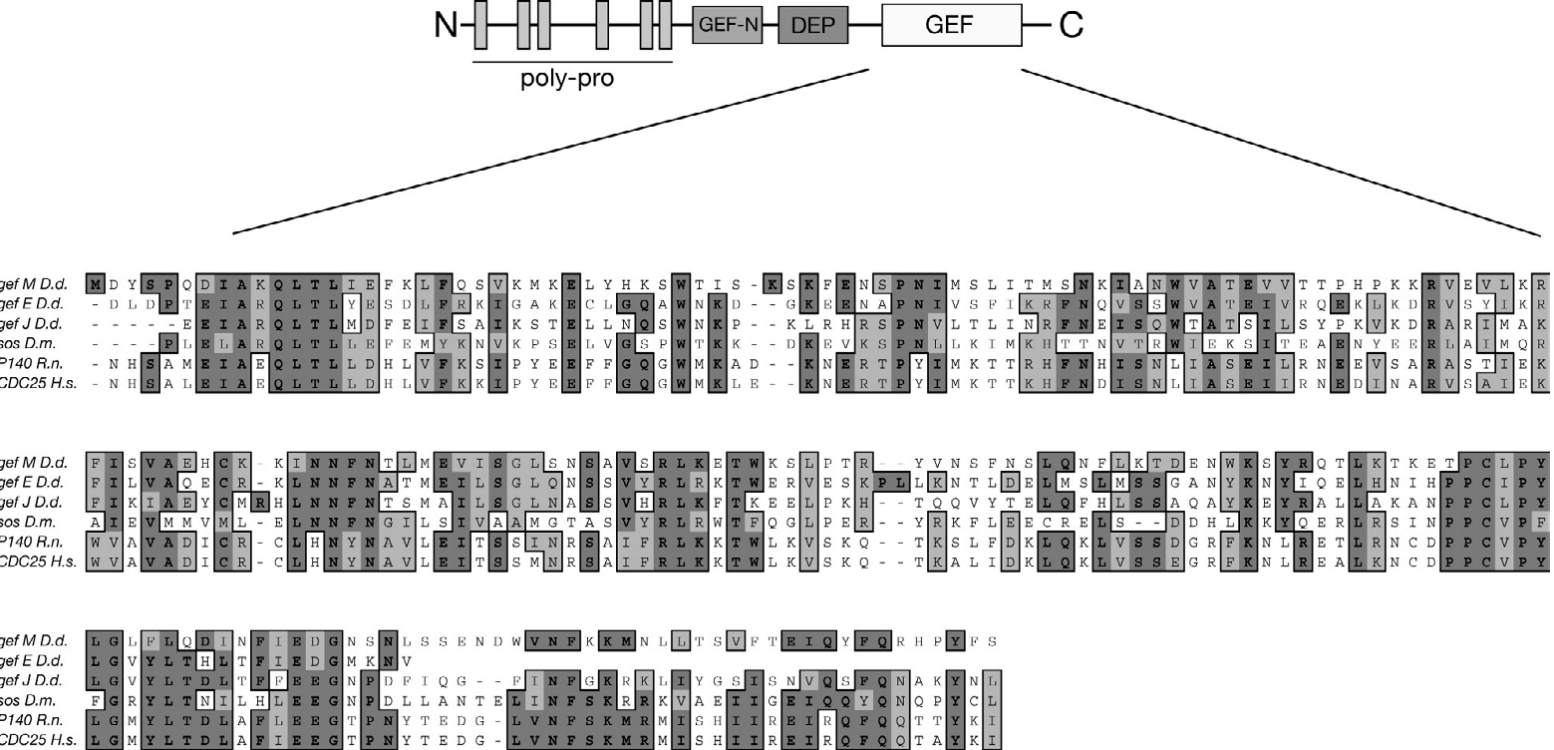
**Schematic representation of the different functional domains of RasGEFM identified with MotifScan**. The RasGEFM protein contains the following recognizable modules: 6 proline rich regions, a RasGEF N-terminal domain, a DEP domain and a RasGEF catalytic domain. The sequence of the RasGEFM catalytic domain (aminoacids 673–863) has been aligned, using the ClustaIW program, with representative RasGEFs proteins from different organisms and others putative *Dictyostelium *RasGEFs. Identical and conserved aminoacid residues are boxed in dark or light gray respectively. Accession numbers are referred to GeneBank: [AAN46882 *D.d*.GEF M, AAN46874 *D.d*.GEF E, AAN46879 *D.d*.GEF J, A41216 *D.m*.Sos, P28818 *R.n*.p140, A38985 *H.s*. CDC25]

To identify Ras regulator proteins in the *Dictyostelium *genome a bioinformatic approach was taken, based on a tBLASTn algorithm, to search the *Dictyostelium *genome project databases [13] and [14]. Using known RasGEFs domains as query, we identified approximately 30 genes encoding for proteins with significant homology to putative RasGEF proteins (unpublished results). Among the putative RasGEF encoding genes, one of these, designated as *Dd rasGEFM*, was isolated for functional studies. The *rasGEFM *gene, located on the chromosome 2, is organized in 4 exons, which are interrupted by 3 introns. Southern blot analyses performed under high and low stringency conditions, and extensive analysis of the *Dictyostelium *genomic sequence databases, indicated that the gene is present as single locus (data not shown). The *rasGEFM *gene encodes a relatively large multi-domain protein of 929 amino acids, with a calculated molecular mass of approximately 102 kDa, whose domain structure is displayed in Fig. 1. The amino-terminal region of the protein contains six short poly-proline stretches, which are putative consensus docking site for SH3 or WW domains [15] whereas the carboxy-terminal region possesses the putative RasGEF catalytic domain. BLAST analysis shows that the catalytic domain, with evolutionary identical amino acid residues conserved, shares high homology with the murine p140GRF (30% identity, 59% homology). Among *Dictyostelium *RasGEF proteins, the most similar to RasGEFM are two putative uncharacterized RasGEFs (RasGEFE and RasGEFJ) (Fig. 1).

Between the catalytic domain and the amino-terminal poly-proline rich region, there are a DEP and a RasGEF-N terminal domain. The RasGEF-N terminal module is peculiar only for Ras specific GEFs, and it is likely to have a purely structural role [16]. The DEP (Dishevelled-Eglin-Pleckstrin homology) domain, located between the catalytic and the RasGEF-N terminal domain, shows significant homology to the DEP domain of the human pleckstrin 25% overall homology and 11% identity, versus a 27% overall homology and 14% identity displayed by the *Dictyostelium *GbpC DEP [11] when compared to the human plekstrin counterpart. DEP is a widespread motif found in proteins involved in Wnt signalling, in regulators of G protein signalling (RGS), in pleckstrin and other signalling proteins [17,18], responsible either for targeting proteins to the membrane or mediating protein-protein interaction, although the underlying molecular mechanism remains still unknown.

### *rasGEFM *gene expression is developmentally regulated and partially controlled by the G protein

**Figure 2:**
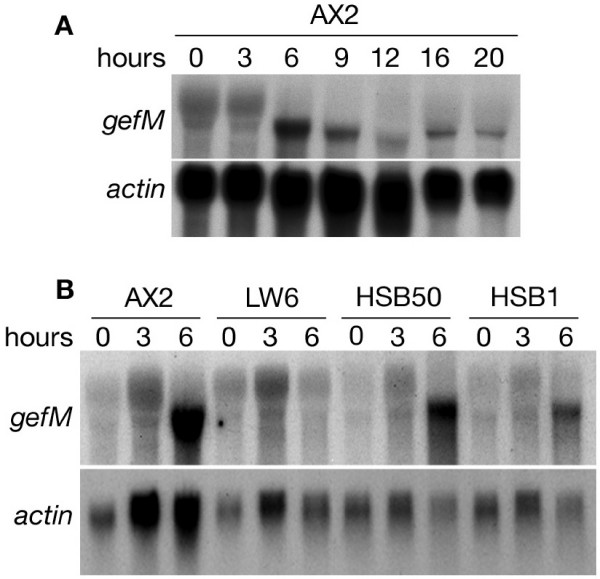
**Northern blot analysis of *rasGEFM *expression in parental and mutant strains**. (*A*) Total RNA was extracted from AX2 cells developed in suspension for 0 to 9 hours or on filters. In the latter case the cells were harvested at mound (12 hours), first finger (16 hours) and preculminant (20 hours) stages. The membrane was hybridized to a radiolabelled *rasGEFM *specific probe (probe *a*) corresponding to bp 760–1518 of the cDNA clone and to the *actin *gene used as a loading control. (*B*) Total RNA was extracted from different developmental *Dictyostelium *mutants starved in shaking suspension up to 6 hours. LW6 (G protein β subunit minus), HSB1 (PIA *ts*-mutant, defective in the G-protein adenylyl cyclase activation) and HSB50 (mutant blocked at mound stage).

One of the prominent features of *Dictyostelium *life cycle is the transition from solitary amoebae to multicellular aggregates. This transition is triggered by starvation of the cells, is enhanced by periodic release of cAMP, and results in the coordinated activation and repression of aggregation-specific and growth-phase genes, respectively [19]. We examined the expression pattern of the *rasGEFM *gene during development of wild-type cells and in three mutant strains, which are blocked at sequential steps of development. Two transcripts were detected in Northern blots: the upper one is present during growth and at the beginning of development and disappears at 6 hours of starvation. The lower transcript is barely visible at the beginning of starvation, reaches its maximal expression at 6 hours of development and decreases thereafter, though being present up to the end of development (Fig. 2A).

We examined *rasGEFM *expression in the LW6 mutant lacking the β subunit of heterotrimeric G protein [20]. Although two Gβ genes are present in the *Dictyostelium *genome, disruption of one of them blocks all G protein-dependent pathways in LW6 mutant cells, which fail to respond to and to relay cAMP, to express aggregation specific genes and to develop. In this strain only the *rasGEFM *upper transcript is detected during starvation, while the lower transcript fails to accumulate (Fig. 2B). This indicates that up-regulation of the lower transcript is under control of the heterotrimeric G protein. Gene expression was also analyzed in the HSB1 and HSB50 mutants. HSB1 is defective in cAMP relay, due to a temperature-sensitive mutation in the adapter protein PIA, which is essential for adenylyl cyclase activation [21,22]. HSB1 cells thus fail to aggregate but, in contrast to LW6, express at moderate level aggregation-specific genes and are sensitive to exogenous cAMP pulses. The HSB50 mutant undergoes chemotaxis and aggregation, but is blocked at mound stage [23]. In both mutants, the *rasGEFM *expression pattern was similar to that observed in the parental strain (Fig. 2B), suggesting that transcription of the lower transcript does not require cAMP relay, even though it may be enhanced by cAMP pulses, similarly to other aggregation-specific genes [24]. It is worth mentioning that the two transcripts may arise from differential splicing or different degree of polyadenylation. Screening 24 cDNA clones obtained by using mRNA from 6-h starved cells resulted in a signle sized cDNA to be present. Sequencing three such clones gave rise to a transcript containing all four exons. Assuming this transcript to correspond to the most abundant mRNA species at this time point, namely the lower band, then we should conclude that differential splicing is unlikely and the upper band is the result of extensive polyadenylation. Additional experiments are required to confirm this hypothesis and to understand this intriguing developmentally regulated changes in *rasGEFM *gene expression.

### *rasGEFM *null mutant is delayed in the acquisition of aggregation competence and blocked at the aggregation stage

**Figure 3:**
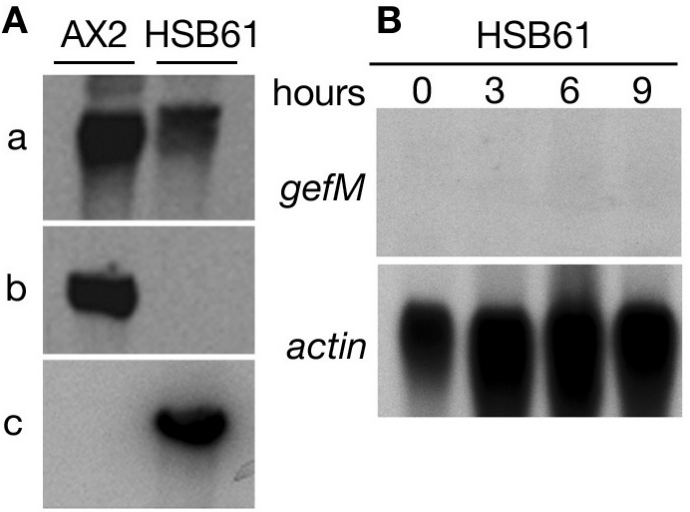
**Disruption of the *rasGEFM *gene**. (*A*) Southern blot of genomic DNA from parental strain (AX2) and *rasGEFM *null mutant (HSB61). Genomic DNA was digested with *Eco*RI, separated in 0.8% agarose gel, blotted onto nylon membrane and probed with probe *a *(*rasGEFM *N-terminal fragment corresponding to bp 0–617 of the cDNA clone), probe *b *(corresponding to bp 760–1518 of the *rasGEFM *cDNA clone) and probe *c *(*bsr *cassette). Three different probes were used to characterize the genomic locus of the mutant and the parental strain. In the *rasGEFM *null strain the central part of the gene (recognized by probe *b*), was replaced by the blasticidin cassette (recognized by probe *c*), which has the same size of the replaced fragment. Because of that, the size of the locus remains unchanged, but the central part is recognized specifically by *bsr *(probe *c*) or probe *b *in HSB61 or AX2, respectively. Both AX2 and HSB61 genomic loci are recognized by the probe *a*. (*B*) Northern blot of total RNA extracted from HSB61 cells at the indicated time points of growth and development. The membrane was hybridized to the *rasGEFM *and to the *actin *gene.

**Figure 4:**
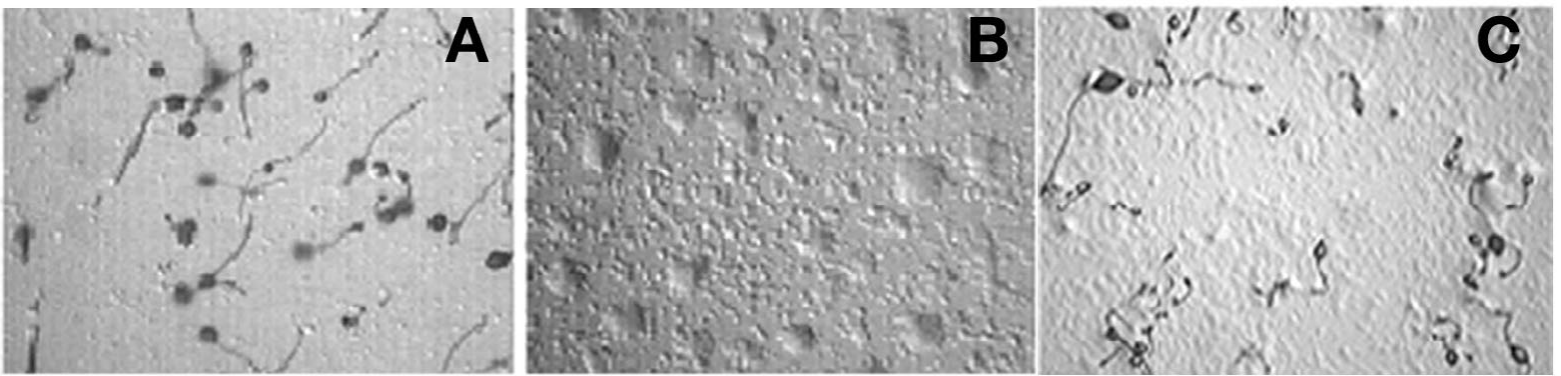
**Developmental phenotypes of (*A*) wild-type or (*B*, *C*) *rasGEFM *null cells**. (*A*, *B*) AX2 or HSB61 cells were plated at the beginning of starvation at a concentration of 1 × 10^7^cell/ml on non-nutrient agar (approx. 6.5 × 10^5 ^cell/cm^2^). (*C*) HSB61 cells were pulsed with cAMP for 10 hours before plated on agar. The final phenotype after 24 hours is shown.

To gain insight into the function of *rasGEFM*, the gene was inactivated by homologous recombination. Disruption was confirmed by Southern and Northern blot analysis of the mutant strain, named HSB61 (Fig. 3). HSB61 cells grew normally both in axenic medium, or on bacterial lawn, but were defective in development. In contrast to the parental cells (Fig. 4A), which form fruiting bodies after about 24 hours of starvation, HSB61 cells formed small, flattened aggregates, unable to develop into tipped tight mounds (Fig. 4B). Aggregate formation was density-dependent. If cells were plated on non-nutrient agar at concentration of 1 or 2 × 10^5^/cm^2 ^no aggregates were formed, whereas control cells formed aggregates. At 1 × 10^6 ^cells per cm^2 ^the mutant cells formed small aggregates, similar to those shown in Fig. 4B, but many cells failed to aggregates. Treating cells with cAMP pulses rescued in part the mutant phenotype; if aggregates, formed in suspension after 6–8 hours pulses, were transferred on agar, they continued to develop and to form small fruiting bodies, although many cells failed to aggregate (Fig. 4C). We have failed to observe cells forming streams, even in 6 to 8 hours pulsed cells, although elongated cells very near to aggregates are found, suggesting that some short-range chemotaxis may occur.

**Figure 5:**
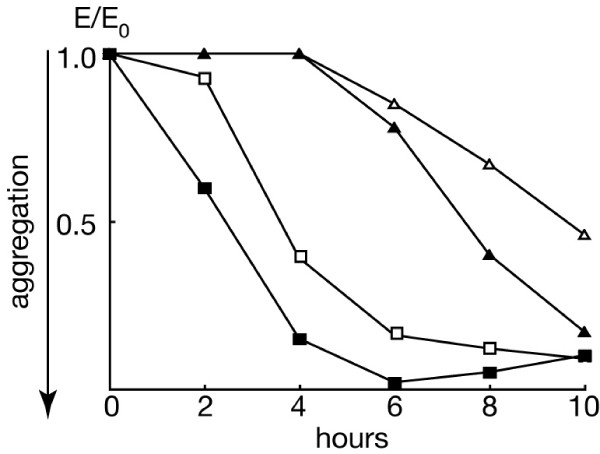
**Appearance of EDTA-stable contacts in (*open symbols*) untreated and (*closed symbols*) cAMP treated cells**. Cells of (*squares*) AX2 or (*triangles*) HSB61 were developed in suspension. At the developmental time indicated in the abscissa cells were taken, incubated with 10 mM EDTA and cell adhesion measured in the agglutinometer of Beug and Gerisch, as described in Methods. A representative experiment is shown.

The finding that HSB61 formed flat aggregates prompted us to test whether the mutant was defective in cell-cell adhesion or chemotaxis. Intercellular adhesion in *Dictyostelium *is developmentally regulated, as growth-phase cells are weakly adhesive, and this adhesion is completely blocked by EDTA. During the first 5–6 hours of development, cells express the adhesion glycoprotein csA on the cell surface, which is responsible for an EDTA-resistant form of adhesion (for a review see [25]), and at least in part, for post-aggregative pattern formation [26]. We tested the ability of HSB61 cells to develop EDTA-resistant adhesion over the first 8 hours of development. As shown in Fig. 5, HSB61 cells exhibited a delay of 5 to 6 hours in the appearance of EDTA-resistant adhesiveness compared to the parental strain. Cell treatment with cAMP pulses, which are known to stimulate expression of csA as well as several other aggregation-specific genes [19], accelerated acquisition of EDTA-stable adhesion, though in contrast to the parental strain the mutant cells appeared to be refractory to the pulses for at least 4 hours (Fig. 5).

To examine whether *rasGEFM *null cells were impaired in their ability to chemotax towards cAMP, a micropipette-based assay was used. HSB61 cells resulted strongly impaired in chemotaxis, as will be described further below, but their behaviour improved significantly when pulsed with cAMP. Taken together these results suggest that HSB61 cells fail to aggregate properly, due to impaired periodic cAMP signalling, which is required for optimal expression of aggregation-stage specific genes.

**Figure 6:**
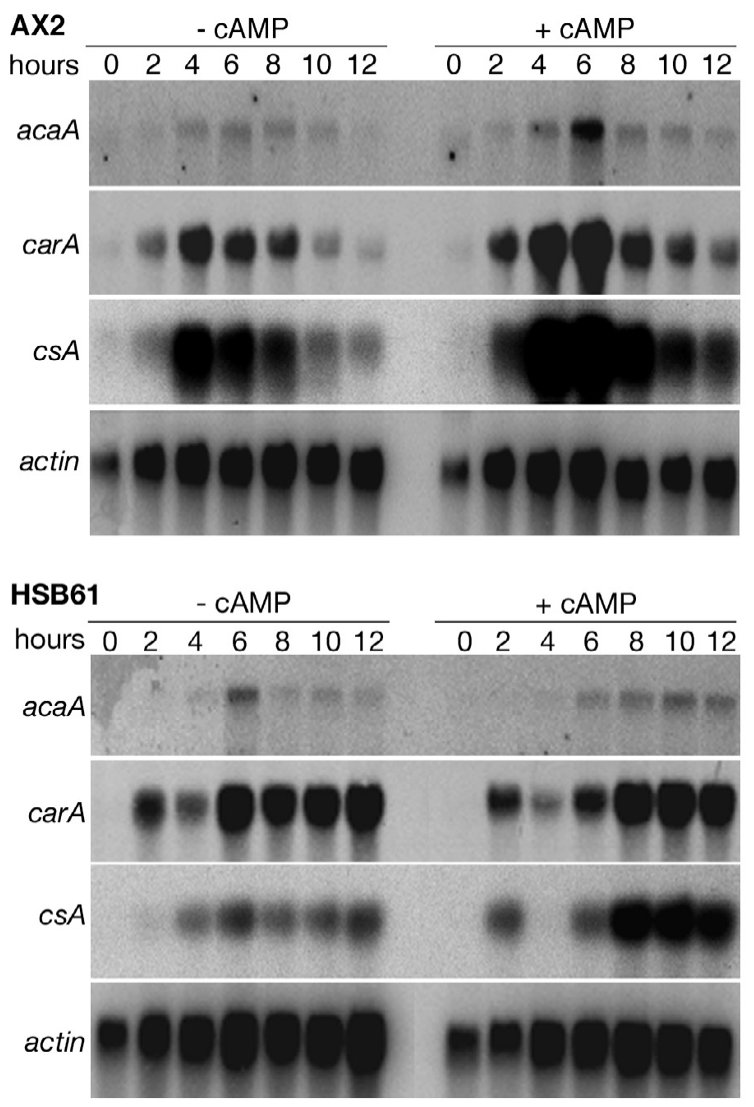
**Early developmental gene expression in AX2 and HSB61 cells**. Total RNA was extracted from cells pulsed (+ cAMP) or not (- cAMP) in suspension for the time indicated. After electrophoresis and transfer, the membranes were hybridized with radiolabelled *acaA*, *carA*, *csA*. *Actin *was used as control.

To confirm this hypothesis, we followed the expression of three such genes, namely *carA*, *acaA *and *csA*. *CarA *and *acaA *encode the cAMP receptor cAR1 and adenylyl cyclase A, respectively, whereas *csA *is, as mentioned above, the gene for the cell adhesion glycoprotein csA. Expression of *acaA*, *csA*, and to a lesser extent *carA*, was deranged in the mutant compared to the parental strain. The peak of expression for all three genes was reached in the parental strain between 4 and 6 hours of development followed by down-regulation as cells undergo aggregation. Pulsing with cAMP accelerated the kinetics of their expression, leading to a higher mRNA accumulation rate (Fig. 6 upper panel). In the *rasGEFM *mutant, mRNA expression was delayed and down-regulation was not detected even after 12 hours starvation. Cyclic AMP pulses elicited a stimulatory effect, though not as efficient as in the parental strain (Fig. 6 bottom panel).

### Cyclic AMP receptor and G protein dependent activation of adenylyl and guanylyl cyclases is altered in *rasGEFM *null cells

The finding that the HSB61 mutant was delayed in the acquisition of the aggregation competence but partially responded to cAMP pulses suggested that the RasGEFM protein might be involved in cAMP receptor-mediated signaling. We therefore monitored cAMP accumulation during the early hours of development and tested adenylyl and guanylyl cyclase activities *in vitro*.

**Figure 7:**
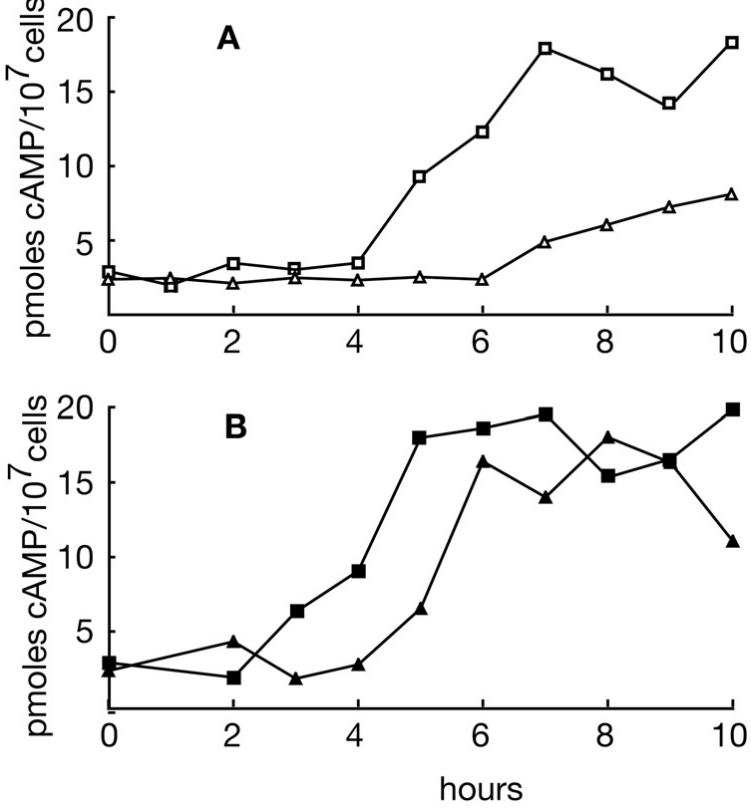
**Cyclic AMP accumulation in AX2 and HSB61 during development in shaken suspension**. (*Squares*) AX2 or (*triangles*) HSB61 cells were incubated in suspension for the time indicated in the abscissa. (*A*) control cells or (*B*) cells treated with cAMP pulses every 6 minutes. At the time indicated, cell aliquots were quenched with perchloric acid, neutralised with KOH, and total cAMP in the samples was determined by radioimmunoassay as described in Material and Methods.

In AX2 cells, the basal cAMP is low at the beginning of development and rises sharply at around 4 hours of development, reaching a maximum by around 7–10 hours, after which it decreases (Fig. 7A) [19,24]. In HSB61 cells, cAMP started to accumulate with a delay of 2 hours and increased at a very low rate, reaching at 10 hours of starvation less than half the maximal concentration of the wild type (Fig. 7A). Cell treatment with cAMP pulses accelerated of two hours endogenous cAMP accumulation both in wild type and the mutant, restoring almost normal levels of cAMP in the latter (Fig. 7B).

**Figure 8:**
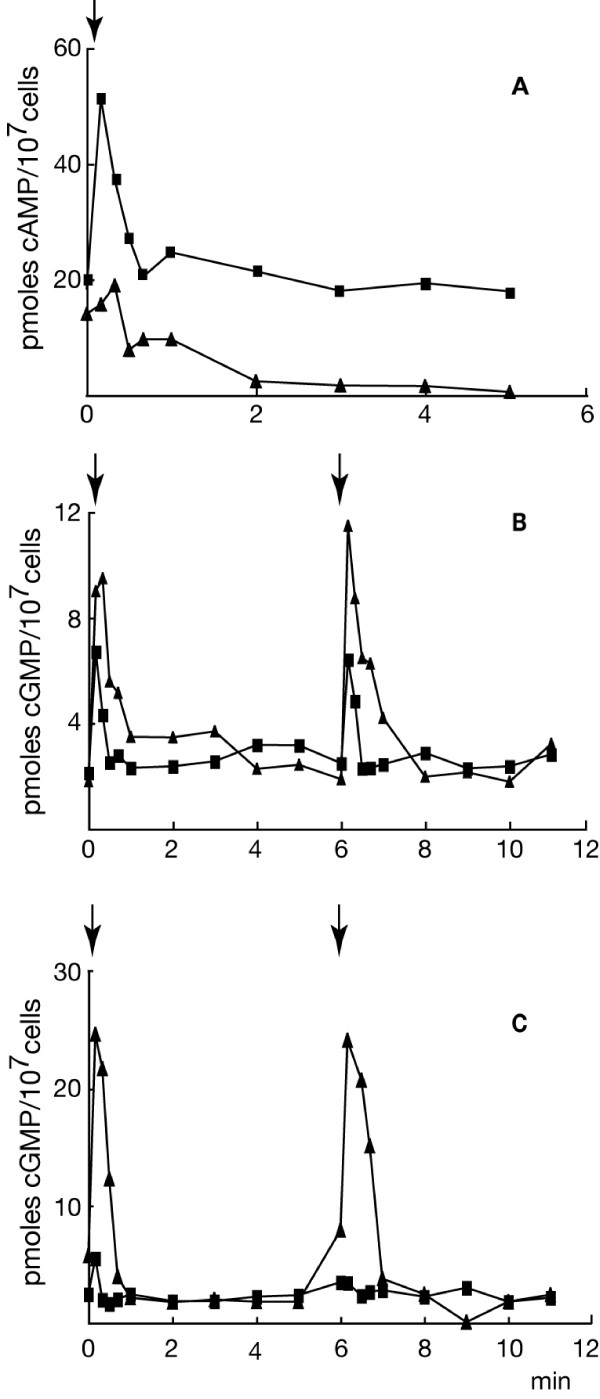
**Cyclic AMP stimulation of (*A*) adenylyl and (*B*, *C*) guanylyl cyclase activity**. (*Squares*) AX2 or (*triangles*) HSB61 cells were treated with cAMP pulses for (*A*, *B*) 5 or (*C*) 9 hours. In conjunction with a cAMP pulse (arrows), samples were taken at the time indicated in the abscissa for determining total concentration of (*A*) cAMP or (*B*, *C*) cGMP. Cyclic-nucleotides were measured using the radioimmunoassay kit as described in the section Material and Methods. Representative experiments are shown.

Cyclic AMP accumulation in the pre-aggregative and aggregation stage results from the periodic activation of adenylyl cyclase, which is stimulated in autocrine and paracrine loops by cAMP. This leads to both increased accumulation of the enzyme and oscillatory stimulation of its activity, with a period of about 6 minutes [19]. Exogenously supplied cAMP pulses mimic the endogenous oscillations of cAMP and give insight on potential defects in cAMP relay or other cAMP induced responses in mutant cells. We investigated cAMP and cGMP changes in HSB61 cells during a period of cAMP pulses. As shown in Fig. 8A, in response to a cAMP pulse, 5 hours starved and cAMP-treated HSB61 cells displayed a dramatically reduced increase in cAMP compared to AX2 cells. In contrast the cGMP peak in the mutant was about twice that observed in the parental strain (Fig. 8B). Remarkably, when cells were assayed after 9 hours pulsing, cGMP peaked 5 to 10 fold higher compared to AX2 cells (Fig. 8C), while the cAMP level increased slightly compared to t5 mutant cells (data not shown). The *rasGEFM *null mutant, therefore, displays significantly increased cGMP and reduced cAMP responses in the pre-aggregative stage and during aggregation compared to the parental strain. The HSB61 developmental defects are supported by light scattering measurements of cells in suspension, which showed that the mutant cells failed to undergo spontaneous light scattering oscillations. When pulsed with cAMP for several hours, light scattering changes were induced, but the responses were lower than in the wild type (data not shown).

**Figure 9:**
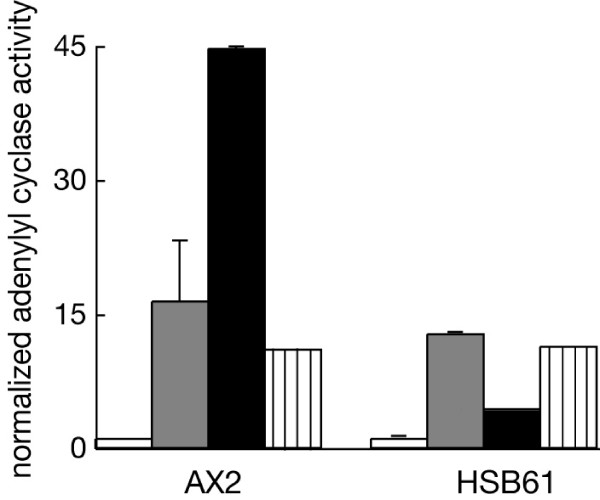
**GTPγS and 2'-deoxy-cAMP induced adenylyl cyclase activation**. AX2 or HSB61 cells were pulsed with cAMP for 5 or 7 hours, respectively. Cell lysates were prepared in the presence of (*grey bars*) GTPγS, (*black bars*) 2'-deoxy-cAMP, (*black stripes*) MnCl_2 _and assayed for adenylyl cyclase activity. Plotted values were normalized relative to the (*open bars*) unstimulated activity obtained in the absence of GTPγS or 2'-deoxy-cAMP (0.7 pmol/mg/min and 0.6 pmol/mg/min for AX2 and HSB61 respectively). Values for AX2 and HSB61 cell lysates are the means ± sd of two indipendent experiments run in duplicate.

**Figure 10:**
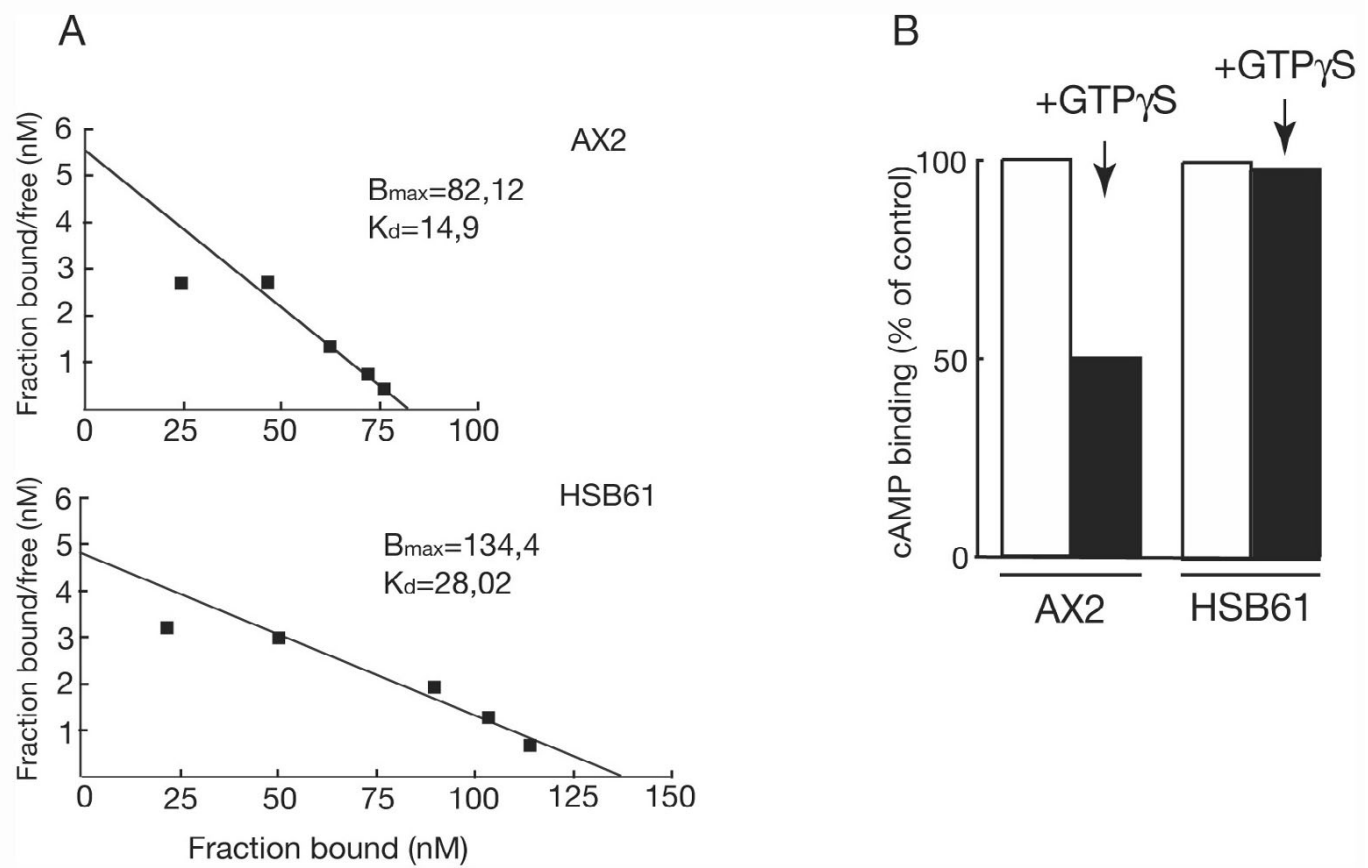
**cAMP binding to the cell surface and its inhibition by GTPγS**. (*A*) Scatchard analysis of cAMP binding in AX2 and HSB61 cells pulsed with cAMP for 5 or 7 hours, respectively. The [^3^H] cAMP binding was determined over a range of 700–19700 nM cAMP by incubation for 5 min at 0°C. A fitted line, B_max _and K_d _are shown in each panel. (*B*) Inhibitory effect of GTPγS (*black bar*) on the binding of cAMP to their cognate receptors. Crude membranes were incubated with [^3^H] cAMP in the absence or presence of GTPγS. Values are indicated as the percentage of cAMP binding in treated normalized to the untreated membranes (*white bar*). In wild type strain cAMP binding dropped from 10.4 ± 0.3 nM to 5.26 ± 0.04 nM for GTPγS untreated and treated membranes, respectively. HSB61 strain displayed comparable absolute values: 16.23 ± 0.25 and 16.00 ± 0.3 nM for GTPγS untreated and treated membranes, respectively. Means of three independent experiments, each run in duplicate, are shown.

The finding of a reduced cAMP response in the mutant prompted us to assay adenylyl cyclase activity in cell lysates upon stimulation of the cells with 2'-deoxy-cAMP or the slowly hydrolyzable GTP analog GTPγS. The assay was done with mutant cells pulsed with cAMP for 7 hours, since after such treatment the mutant cells accumulated cAMP to levels comparable to 5-h treated control cells, as shown in Fig. 7B. GTPγS stimulated adenylyl cyclase activity about 16 and 13 fold in AX2 and HSB61 cells, respectively (Fig. 9). In contrast, stimulation with 2'-deoxy-cAMP increased the level of cAMP 4 fold in the mutant compared to 44 fold for AX2 cells (Fig. 9). The finding that GTPγS stimulated adenylyl cyclase to about the same level in both strains suggests that basal adenylyl cyclase activity is roughly comparable in mutant and parental strain, and this was confirmed by assaying adenylyl cyclase activity in the presence of Mn^2+ ^(Fig. 9). The strongly reduced adenylyl cyclase activation by 2'-deoxy-cAMP in the mutant could be due to a lower number of cAMP receptors expressed on the cell surface. To exclude this we performed cAMP binding experiments in both cell lines AX2 and HSB61 pulsed for 5 and 7 hours, respectively. The total amount of cAMP receptors was comparable, as both maximum amount of ligand bound to the receptor (Bmax), and dissociation constant (K_d_) were in the same range in wild type and mutant cells (Fig. 10A).

These data strengthen the hypothesis that the strongly reduced adenylyl cyclase activation in HSB61 cells is G protein independent, and suggest that Ras GEFM may be located upstream of the heterotrimeric G protein and downstream of the cAMP receptor. If this hypothesis is correct, cAMP binding to membranes upon treatment with GTPγS should be altered in the mutant compared to the wild type, since the affinity of cAMP receptors differs when they are complexed with G proteins [27]. Consistent with this hypothesis, cAMP binding to HSB61 membranes was not affected by GTPγS, whereas it was inhibited cAMP binding approximately 50% in wild type membranes (Fig. 10B).

Taken together, these results lead us to conclude that Ras GEFM very likely acts between the cAMP receptor and the heterotrimeric G protein.

### RasGEFM is not the activator of RasC or RasG

**Figure 11:**
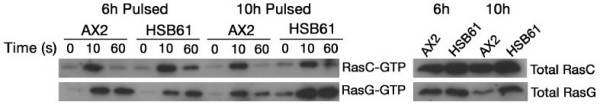
**RasC and RasG activation in HSB61 mutant**. AX2 or HSB61 cells were pulsed with cAMP for the time indicated, concentrated ten times, treated with cAMP and immediately lysed. The lysates were incubated with GST-Byr2 (RBD) as described in Material and Methods. The precipitate was subjected to electrophoresis and Western blot and hybridized with antibodies against RasC or RasG. Time (*in seconds*) after cAMP treatment is indicated. Total RasC and RasG in cell lysates from AX2 or HSB61 are shown on the right.

Two previously characterized Ras proteins, RasC and RasG, have been shown to be involved in cAMP mediated signalling events [28-30]. Therefore, it was tempting to hypothesize that RasGEFM could function as the putative exchange factor for either of these Ras proteins. Activated Ras can be measured by using a Ras Binding Domain (RBD) to affinity purify Ras proteins. Given that the RBD-Ras interaction is dependent on Ras being in a GTP bound form, one can selectively measure activated Ras from cellular lysates. An assay has recently been described for *Dictyostelium *Ras proteins employing the RBD from *S. pombe *Byr2, and it has been shown that both RasC and RasG are transiently activated by cAMP [30]. To determine whether RasGEFM mediates this activation, we challenged 6 hours pulsed AX2 and HSB61 cells with cAMP and observed the kinetics of Ras activation (Fig. 11). While the levels of both activated RasC and RasG increased upon cAMP stimulation in HSB61, the maximum level of RasG-GTP was greatly reduced relative to AX2, whereas RasC activation was largely unaffected in the mutant. This suggested the possibility that RasGEFM may be partly responsible for mediating the activation of RasG. However, when 10-hour pulsed cells were stimulated with cAMP, RasG activation was restored. Interestingly, the total level of RasG remained at a very high level relative to AX2. This data shows that RasGEFM is unlikely to be an activator of RasC or RasG, and the partial loss of RasG activation seen in 6 hours cells may be a secondary effect of reduced gene expression. In addition, the finding that RasG is activated after prolonged cAMP treatment favours the notion that a developmentally regulated component, regulated by cAMP signalling, is directly or indirectly required for RasG activation.

### Ca^2+ ^influx in *rasGEFM *null mutant is reduced

In addition to the effects on cAMP and cGMP levels, the second messenger Ca^2+ ^is rapidly regulated by cAMP binding to the receptor [31]. The finding that chemotaxis and cGMP response were altered in the mutant, prompted us to test chemoattractant-induced Ca^2+ ^entry in HSB61 cells. Ca^2+ ^entry is negatively regulated by cGMP [32], and both Ca^2+ ^and cGMP have been implicated in regulating chemotactic motility [33].

**Figure 12:**
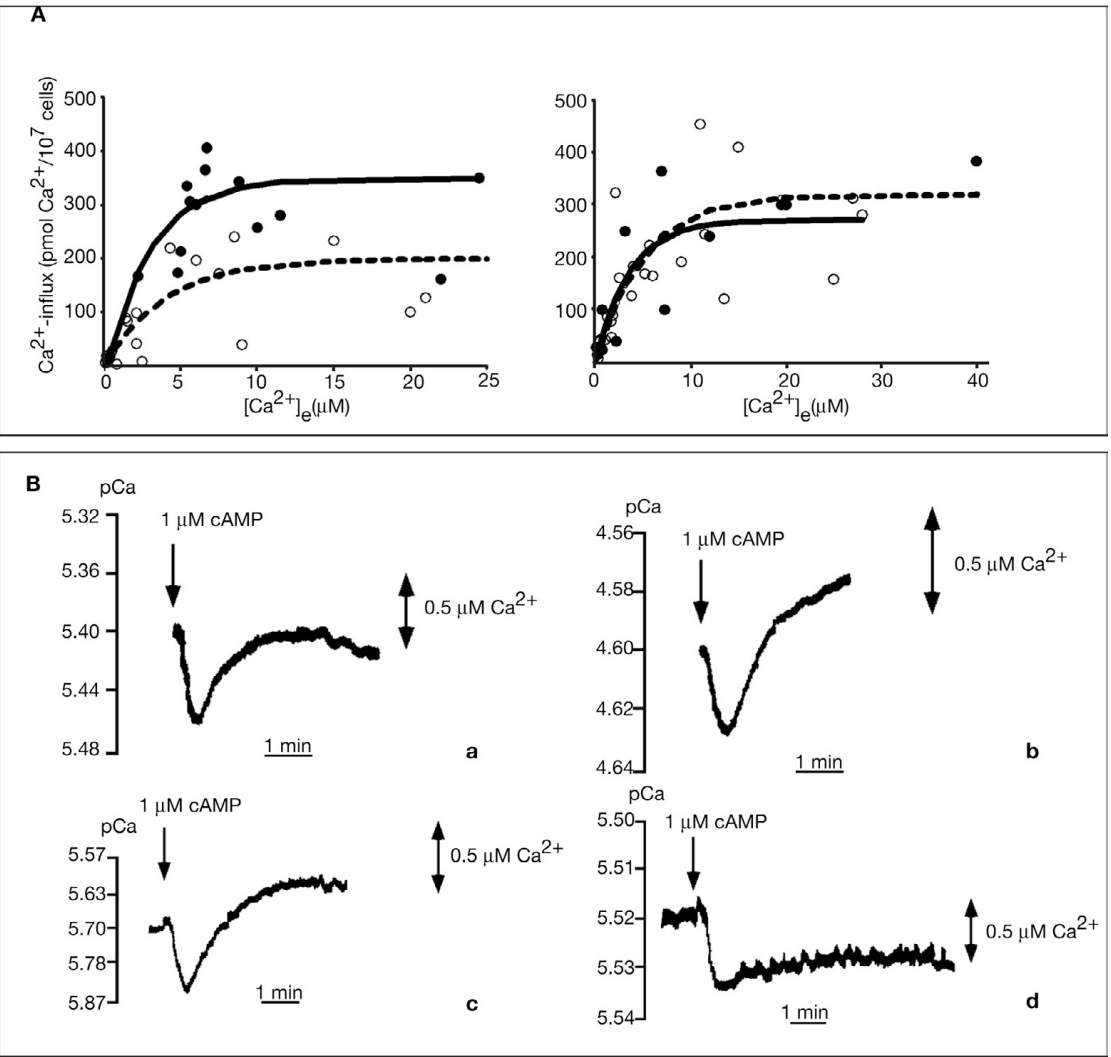
**Ca^2+ ^influx in HSB61 mutant**. *A*) Dependence of cAMP-induced Ca^2+^-influx from [Ca^2+^]_e _measured in unpulsed and pulsed HSB61 cell suspensions. Dose-response curves are shown for (*left*) untreated cells and (*right*) cells treated with 20 nM cAMP pulses. In both cases, cells were tested after 4–5 hours starvation. White circles depict the HSB61 cells and black circles the AX2 cells. Data are presented for at least 4 independent experiments for each cell type. *P *values = 0.00068 and 0.8368 for left and right graphs, respectively (Wilcoxon test). *B*) cAMP-induced Ca^2+^-influx in HSB61 and wild-type cells. Single electrode recordings are shown for (*a*, *b*) mutant cells either (*a*) unpulsed or (*b*) pulsed overnight with 20 nM cAMP. For comparison (*c*, *d*) wild-type cells are shown after 5 hours of development in (*c*) and after 7.5 hours in (*d*). Addition of cAMP is indicated.

The basal levels of intracellular Ca^2+ ^concentration were comparable in parental and mutant cells and elevation in response to a cAMP pulse was only slightly reduced in the mutant (data not shown). Cyclic AMP-induced Ca^2+^influx depends on the extracellular Ca^2+^concentration and on the dose of the cAMP stimulus. The influx is increased with elevation of the concentration of both parameters [34]. Ca^2+ ^influx is maximal in AX2 cells after 4 to 6 hours of development and remains constant thereafter [34]. We determined maximal Ca^2+ ^influx in unpulsed and pulsed HSB61 cells. As shown in the saturation curve of Fig. 12A, *left*, maximal Ca^2+ ^influx was reduced to about 50% of the control in unpulsed HSB61 cells, but influx was restored when the mutant cells were pulsed with cAMP (Fig. 12A, *right*). Surprisingly, both in unpulsed or cAMP pulsed HSB61 cells, the kinetics of single Ca^2+^response was as rapid as the wild type in terms of influx and efflux (Fig. 12B, *a-b*). The increased concentration level of cGMP in the HSB61 mutant did not affect the timing of Ca^2+ ^influx. This suggests that either cGMP level must be constantly high to affect Ca^2+ ^entry [27] or that cGMP has no effect on Ca^2+ ^influx [35].

As shown in Fig. 12B*d*, AX2 cells at 8 hours of development, which corresponds to late aggregation stage, displayed a rapid Ca^2+ ^influx in response to cAMP, followed by a very slow efflux. Later on in development, no efflux could be detected [34]. In HSB61 cells, even when pulsed for as long as 12 hours, the kinetics of efflux was similar to 4–6 hours AX2 pulsed cells (Fig. 11B, *b *and *c*). Thus, these data further confirm that the mutant development is delayed even after 12 hours pulsing with cAMP. In addition, the finding that maximal Ca^2+ ^entry in response to cAMP is reduced in the mutant may account, at least in part, for the chemotactic defect of the mutant.

### Mutant HSB61 is impaired in chemotactic, but not in spontaneous cell motility

When cell motility was analysed, differences between mutant and parental strains were only detected during chemotaxis. Spontaneous cell motility was indistinguishable between both cell types. Directional cell migration in response to external chemoattractant gradients implies at least three steps: a) sensing the chemoattractant, b) formation of a leading front and c) cell polarization, with suppression of lateral pseudopods, followed by forward movement and uropod detachment [33].

**Figure 13:**
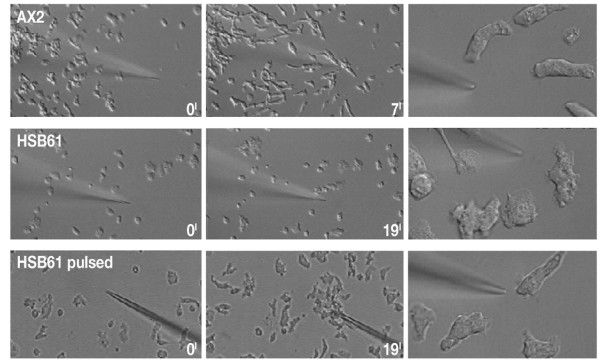
**Chemotaxis of wild-type and HSB61 cells**. Cells were developed in shaken suspension, either in the presence or absence of exogenous cAMP pulses, plated on coverslips and tested for chemotaxis towards a microcapillary diffusing cAMP. Upper and middle panels show AX2 or HSB61 cells starved for 5 hours, bottom panel shows HSB61 cells treated with cAMP pulses for 10 hours (see additional files 1, 2, 3). Higher magnifications of each cell sample are shown on the right. Numbers: time in minutes, starting after positioning of the microcapillary (0' time).

As depicted in Fig. 13 and additional file 1, aggregation competent wild type cells, when challenged with the cAMP-loaded micropipette, become highly polarized, with a clearly defined leading front and a posterior uropod, and rapidly move towards the cAMP source. In their movement, AX2 cells eventually adhere to each other into streams, due to outward relay of the signal (Fig. 13, upper panel). Five hours-starved HSB61 null cells behaved differently: they sensed the gradient and extended a membrane lamella towards the micropipette, but they also extended several lateral pseudopodia with high frequency, thus displaying a severe polarization defect. In contrast to the parental strain, the mutant cells exhibited a rather flattened shape and an apparently increased cell-substratum adhesion. As a result, their chemotactic orientation and motility were strongly reduced (Fig. 13, middle panel and additional file 2). Pulsing with cAMP for at least 6–8 hours partially rescued the mutant cell phenotype, in that these cells were now better polarized and displayed an organized leading front, moving towards the capillary in a way similar to AX2, though the cell population was somewhat heterogeneous in that respect (Fig. 13, lower panel and additional file 3). The chemotactic speed of mutant cells changed from 1.43 ± 0.49 μm/min for untreated cells to 6.2 ± 2.1 μm/min for cAMP pulsed cells. Both values were below the 9.7 ± 1.81 μm/min observed for 5 hours starved AX2 cells.

Cyclic AMP pulses failed to induce cell streaming: the HSB61 cells chemotaxed towards the capillary mostly as single cells, suggesting that spontaneous cAMP relay was still reduced in the mutant.

**Figure 14:**
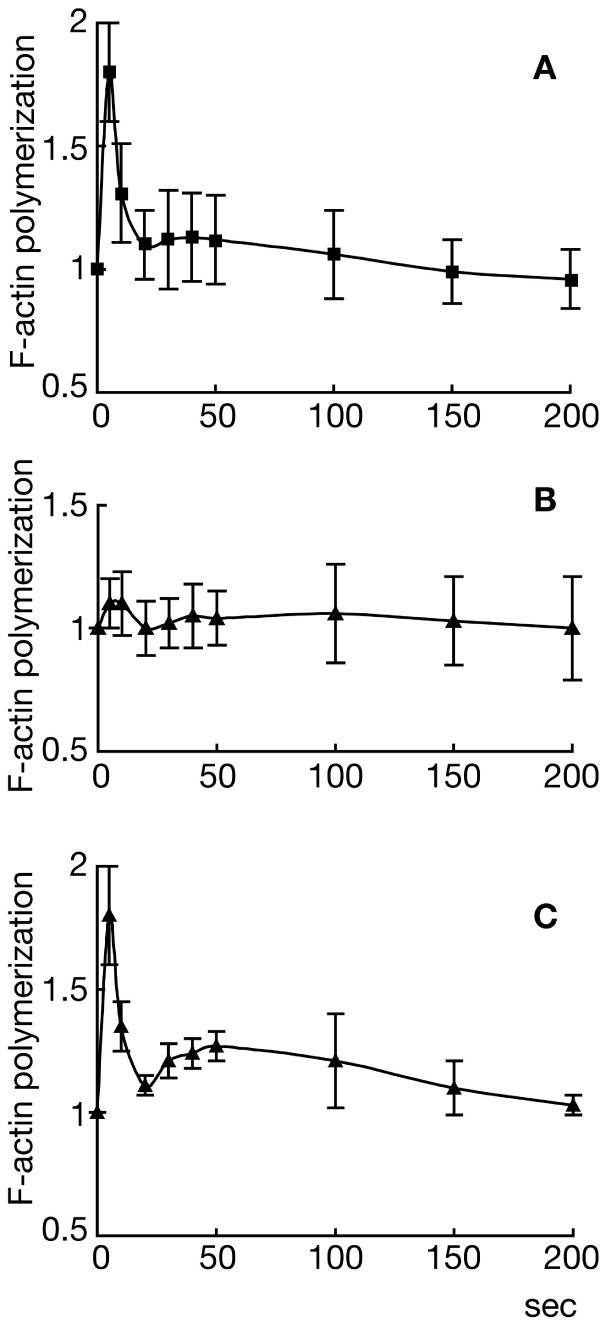
**Actin polymerization assay in response to chemoattractant stimulation**. The cells concentrated at 2 × 10^7^/ml were stimulated with 1.0 μm cAMP (time 0) and the F-actin formation measured with the phalloidin binding assay as described in Material and Methods. Starving (*A*) AX2 and (*B*, *C*) HSB61 cells were treated with cAMP pulses and tested for F-actin polymerization after (*A*, *B*) 4 or (*C*) 6 hours of starvation.

Stimulation with chemoattractants causes polymerization and reorganization of actin, and this has been correlated with the extension of new pseudopods during chemotaxis [36]. We investigated the levels of F-actin in HSB61 cells following cAMP pulses. Wild-type cells showed the expected biphasic response with a sharp peak of F-actin at about 5 seconds after stimulation and a lower second peak at about 50 seconds (Fig. 14A). A very low actin response was detected in HSB61 cells pulsed for 4 hours (Fig. 14B), but when cells were pulsed for at least 6 hours the intensity of actin response was similar to that of the parental strain (Fig. 14C).

## Discussion

In this study we have reported the isolation and characterization of a novel RasGEF, named RasGEFM, which is required for proper *Dictyostelium *development. RasGEFM is peculiar in that it contains 6 poly-proline stretches that represent putative interacting sites for SH3, WW and/or EVH1 containing proteins [37], and a DEP1 motif.

Disrupting *rasGEFM *results in cells to be blocked at the aggregation stage, forming rather small, flattened aggregates that fail to develop further. The *RasGEFM*-null phenotype could be partially rescued by pulsing cells with cAMP. Gene expression studies and functional assays indicate that the mutant phenotype is due to defective developmental expression of cAMP relay and are consistent with a role of RasGEFM in regulating cAMP signalling. The mutant phenotype differs, however, from the phenotype displayed by other mutants that are strictly defective in adenylyl cyclase activation, such as mutants in RasGEFA [9], PIA [21,22] CRAC [38] and RasC [29]. PIA and CRAC are cytosolic proteins that couple the G protein to adenylyl cyclase, and RasGEFA as well as RasC have been shown to also be essential for adenylyl cyclase activation. In contrast to *rasGEFM *null cells, which form small flat aggregates, all these other mutants fail to aggregate, although they respond to cAMP pulses. Their chemotactic motility in response to cAMP diffusing from a microcapillary is also much less impaired, and guanylyl cyclase activation is rather normal and not enhanced as in the *rasGEFM *null mutant.

These findings indicate that these mutants display different alterations in signal transduction downstream of the cAMP receptor. The RASGEFM protein, in contrast to RasGEFA, PIA and CRAC, appears to be located downstream of the cAR1 receptor, but upstream of the G protein, thus possibly affecting both G protein dependent and G protein independent pathways.

*RasGEFM *null cells have defects in both adenylyl cyclase activation and in chemotaxis, characteristics similar to those of *rasC *null cells. It might have been expected, therefore, that RasGEFM would act upstream of RasC. However, we now have direct evidence that RasGEFA directly activates RasC and is the only GEF responsible for the activation of RasC (Kae *et al*, unpublished). Consistent with this result, there was no major effect on the cAMP dependent activation of both RasC and RasG in the *rasGEFM *null cells.

Several attempts have been performed to try to rescue the HSB61 phenotype but so far none of them succeeded, although a RasGEFM GFP-fused protein of the correct size was expressed (data not shown). This observation, combined with the rather peculiar *rasGEFM *mRNA expression, in which two transcripts are detected and regulated independently, suggests that the failed rescue might be due to the necessity for the RasGEFM protein to be expressed in a regulated way and not under the control of a constitutive promoter, such as the actin 15 promoter. Experiments are in progress in this direction and they will be crucial to confirm that the phenotype is due to direct disruption of the *rasGEFM *gene and not to additional detrimental effects of the transformation. The possibility of a second random mutation, which independently of *rasGEFM *disruption could be responsible for the observed phenotype, cannot be excluded but it seems unlikely to us. The GTPγS inhibition of cAMP binding, together with the stimulating effects of GTPγS on adenylyl cyclase, and the rescuing effect of cAMP, clearly locate the mutant defect upstream of the G protein and suggest a single event to be involved.

When *rasGEFM *null cells are challenged with a cAMP filled micropipette, the chemotactic response is impaired. Apparently the cells sense the gradient but they seem incapable of organizing a leading front. Moreover, several lateral pseudopodia are continuously extended and retracted. As a consequence, the cell fails to polarize, maintains a rather flattened shape and moves "spastically" towards the micropipette. If mutant cells are treated with cAMP pulses for at least 6 to 8 hours, chemotactic orientation and speed are improved, and many cells become elongated and move faster towards the micropipette.

Formation of a leading front, acquisition of polarity and suppression of lateral pseudopodia are processes characterized by the redistribution of cytoskeletal components, with F-actin and numerous actin-binding proteins being enriched at the front and myosin II assembled in filaments at the back of cells [33]. These processes are regulated by the balanced action of several sub-cellular components, including second messengers (e.g. Ca^2+^, cGMP) and proteins such as PI3K and PTEN [39]. A mechanism by which localized Ras activation mediates leading edge formation through activation of PI3K and other Ras effectors required for chemotaxis has been recently proposed for *Dictyostelium *cells by Sasaki *et al. *[28].

Cyclic AMP elicits in the mutant a biphasic actin polymerization response comparable to the parental strain, but the absolute peak values are strongly reduced. Strikingly, when mutant cells are pulsed for additional 2–4 hours the actin response is comparable to that of the wild type. These results suggest to us that RasGEFM is not directly involved in mediating a putative Ras-induced actin polymerization. They are instead consistent with the notion that a developmentally regulated component, required for proper actin recruitment in response to chemoattractant, is absent or expressed under a threshold level in the mutant and is induced by prolonged cAMP treatment.

Taken together our findings support a role for RasGEF M in developmental acquisition of chemotactic efficiency and aggregation competence. The proposed localization of Ras GEFM downstream the cAMP receptor lead us to suggest that Ras GEFM and its cognate Ras may act as dissociating components between G protein coupled receptors and the G protein or molecular switches acting in a G protein independent way. A physical interaction between β 1 adrenergic receptors and a RasGEF has been recently reported in mammalian cells [40].

A peculiarity of the *RasGEFM *null mutant is the elevated cyclic GMP response to cAMP stimulation both at early and late starvation times, which suggests a role for RasGEFM as negative regulator of cAMP receptor-induced guanylyl cyclase activation. Cyclic GMP accumulation has been proposed to be regulated by adaptation [41] and to inhibit pseudopodia formation at the back of the cell by inducing myosin filament formation in the cell cortex [42-45]. Myosin filament formation at the presumptive leading front would be counteracted by myosin phosphorylation due to myosin heavy chain kinase A (MHCK-A), which is selectively recruited at the leading front [45]. As a result, a high cGMP concentration leads to improved orientation in a chemoattractant gradient, as shown in mutant cells lacking cGMP phosphodiesterase [46]. *RasGEFM *null cells, challenged with cAMP diffusing from a microcapillary, show, however, reduced orientation and increased lateral pseudopodia, despite a stronger cGMP response to cAMP. It may be argued that cGMP is rapidly hydrolysed in the HSB61 mutant, in contrast to the cGMP phosphodiesterase null cells. However, if cGMP is down-regulated by adaptation, it must be assumed that as long as cells are exposed to a chemoattractant gradient, as occurs when they are stimulated with cAMP diffusing from a microcapillary, adaptation of guanylyl cyclase is switched off [41], and this should lead in *RasGEF M *null cells to constantly higher levels of cGMP. Why is then chemotactic orientation reduced in the mutant?

A possible explanation for this discrepancy is offered by the reduced actin response to chemoattractant. We have suggested that a developmentally regulated component, which is required for actin recruitment at the presumptive leading front, is lacking/down-regulated in the mutant. Strongly reduced actin polymerization at the front would result in impaired translocation of myosin heavy chain kinase A (MHCK-A), which has been shown to require F-actin binding [47]. As a consequence, a high cGMP level in the mutant, as in wild type cells or PDE null mutants, would induce myosin filaments all over the cell, consistent with its proposed role as global inhibitor [33,45], but myosin filaments in the front would not be dissociated by MHCK-A, due to its impaired recruitment. Thus we propose that in the RasGEFM null mutant chemotaxis is inhibited due firstly to altered F-actin polymerization at the presumptive leading front and secondarily to impaired recruitment of MHCK-A.

## Conclusion

All the defects observed in the *rasGEFM *null mutant can be explained by assuming a modulatory role of the RasGEFM close to the cAMP receptor, which regulates guanylyl and adenylyl cyclases. RasGEFM appears to act as a negative regulator of guanylyl cyclase and a positive regulator of adenylyl cyclase. Currently we have no obvious explanation for this opposite activity. It must be kept in mind, however, that in contrast to adenylyl cyclase, which is stimulated by the Gβ subunit of the heterotrimeric G protein, receptor-dependent stimulation of guanylyl cyclase is more complex and less well understood. Several lines of evidence suggest a role for small GTPases in its activation, independently and in addition to the heterotrimeric G protein [48-50]. If RasGEFM and its putative Ras target act as receptor-linked molecular switches, this may lead to differential, albeit opposite, effects on heterotrimeric G-protein-dependent or independent pathways. Identifying the putative RasGEFM regulated Ras may help in understanding these complex transduction pathways.

## Methods

### Cell cultures, growth, and developmental conditions

Wild type strain AX2-214 and *rasGEFM *null mutant, referred as HSB61, were grown either in liquid nutrient medium at 23°C under shaking at 150 rpm [51] or on nutrient agar plates with *Escherichia coli B/2 *[52]. When cultured in liquid medium, HSB61 cells were supplemented with blasticidin (ICN) at a final concentration of 10 μg/ml. For development on solid substratum, cells were grown to a density of 2–3 × 10^6 ^cell/ml, washed three times in 0.017 M Na^+^/K^+ ^Soerensen phosphate buffer, pH 6.0 and, once deposited on non nutrient 1.5% (w/v) agar plates, allowed to develop at 23°C. For development in suspension, cells were incubated at a concentration of 1 × 10^7 ^cell/ml in Soerensen phosphate buffer. For cAMP treatment, pulses of 20 nM cAMP were applied every 6 minutes using a Braun perfusor VI equipped with 10 ml-syringe [53].

### Measurement of EDTA-stable contacts

EDTA-stable contacts were measured as described by using the agglutinometer of Beug and Gerisch [52].

### Chemotaxis assay

Chemotaxis was studied by using the microcapillary assay [54]. Briefly, cells were seeded onto 35 mm glass base dishes (Iwaki) at a density of approximately 1 × 10^5^/cm^2 ^and local stimulation of chemotaxis was obtained by passive diffusion of cAMP from a microcapillary (Femtotips1, Eppendorf), filled with a 1.0 mM solution of cAMP. The microcapillary was positioned with an automated Zeiss micromanipulator and cells observed either with a 20× or with a Neofluar 100x/1.3 oil immersion objective, equipped with DIC filter. Images were captured at an interval of 0.66 sec. and recorded in a Panasonic videorecorder (AG-TL700) with a ZVS-47DE camera (Zeiss) mounted on a microscope (Zeiss, Axiovert HAL100). The recorded time-lapse movies were transferred to a computer using USB Instant Video package (ADS Technologies).

### Actin polymerization assay

Actin polymerization assays were carried out as previously described [36,55]. Briefly, cells were starved at 2 × 10^7^cells/ml for 4 and 6 hours and pulsed with 1 μm cAMP. At the indicated time points, 100 μl samples were taken and transferred to 1 ml of actin buffer (20 mM K_2_PO_4_, 10 mM PIPES, 5 mM EGTA, 2 mM MgCl_2_, 3.7% formaldehyde, 0.1% Triton X-100, 0.25 μM TRITC-phalloidin, pH6.8). After shaking for 1 hour at room temperature, samples were centrifuged for 10 minutes at 16000 g and the resulting pellet was resuspended in 1 ml methanol. After shaking overnight, the amount of F-actin was determined by measuring the fluorescence with a fluorimeter (Kontron SFM 25). Setting: excitation wavelength 540 nm, emission 570 nm.

### Measurement of cAMP-induced Ca^2+^-influx

Net Ca^2+^-influx after agonist stimulation was done as described previously [34,56].

Cells were developed in suspension as described above. For accelerating developmental gene expression cells were pulsed with 20 nM cAMP every 6 minutes, overnight in case of HSB61 cells, and for 2 hours in case of wild-type cells. At appropriate time points the cells were washed in nominally Ca^2+ ^free tricine buffer (tricine pH 7.0, supplemented with 5 mM KCl) and resuspended at a cell density of 5 × 10^7 ^cells/ml. The cell suspension was then stimulated with cAMP and Ca^2+^-influx measured with a Ca^2+^-sensitive electrode (Möller) and a voltmeter (Metrohm). Statistical analysis was performed with "Wilcoxon test". Light scattering measurements of cells in suspension were done has described by Gerisch and Hess [57]. The cell suspension (2 × 10^7^cell/ml) was aerated in a cuvette and extinction was concomitantly monitored at 500 nm in a Zeiss PM6 spectrophotometer.

### *In vitro *stimulation of adenylyl cyclase

Adenylyl cyclase in *Dictyostelium *lysates was assayed in the presence of 2'-deoxy-cAMP or GTPγS as described by Lilly and Devreotes [58]. Briefly starving cells were treated with 20 nM cAMP pulses every 6 min for different hours. A total of 1 × 10^8 ^cells were pelleted and resuspended in 1 ml of Soerensen phosphate buffer. An equal volume of ice cold lysis buffer containing either 4 mM MgCl_2 _or 4 mM MnCl_2_, 20 mM Tris pH 8.0 was added. Cells were lysed by passage through a 3-μm pore size Nucleopore membrane in the absence or presence of 30 μM GTPγS or 50 μM 2'-deoxy-cAMP and the lysates incubated on ice for 5 min. A 40 μl aliquot of cell lysate was added to a 40 μl assay mix (20 mM DTT, 1 mM ATP, 2 mM MgCl_2 _or 2 mM MnCl_2 _in 10 mM Tris pH 8.0, 0.4 mM IBMX) and incubated at 20°C for 5 min.

The reaction was stopped, by adding 40 μl 0.1 M EDTA pH 8.0 and boiling the sample for 2 min. The total concentration of cAMP in the samples was determined by using the "Biotrak cAMP assay Kit" according to manufacturer's instructions (Amersham Pharmacia Biotech).

### cAMP binding assays

Binding of cAMP to cell surface receptors was determined as previously described by Van Haastert [59]. Briefly, wild-type and mutant cells were starved by shaking in Soerensen phosphate buffer, without or with cAMP pulses for 5 or 8 hours respectively, washed and resuspended at a density of 1 × 10^8 ^cell/ml.

Aliquots of 80 μl of cells were incubated with a radioactive binding mixture, containing 300 nM of [^3^H]cAMP (Amersham Pharmacia Biotech), 50 mM dithiothreitol in 90% saturated ammonium sulphate, and a variety of cAMP concentration ranging between 700 to 19700 nM. Specific binding was obtained by subtracting non specific binding determined in the presence of 1 mM cAMP.

After 5 min. incubation at 0°C, cells were collected by centrifugation at 14000 × g for 2 min, the pellet resuspended in 100 μl of 0.1 M acetic acid and dissolved in 1.3 ml scintillation fluid. Scatchard plots of cAMP binding were done with GraphPad software (GraphPad Inc.).

### GTP-inhibition of cAMP binding to plasma membranes

The assay was performed as described earlier [27]. Brieflly, GTP-inhibition of cAMP binding was measured in a total volume of 100 μl containing PB (10 mM KH_2_PO4/Na_2_HPO_4 _pH 6.5), 5 nM [^3^H] cAMP, 10 mM dithiothreitol, GTPγS (300 μM when present) and 70 μl membranes. Samples were incubated 5 min. at 0°C, centrifuged for 2 min. at 14000 × g, the supernatant was aspirated and the pellet dissolved in 100 μl acetic acid. Radioactivity was determined after the addition of 1.3 ml of liquid scintillation.

### Cyclic AMP and cGMP determination

For the determination of cyclic nucleotides, cells aliquots were taken at different time points before and after a cAMP pulse, and quenched with 1 vol of 2 N perchloric acid [24]. After centrifugation, neutralization of the supernatant with potassium carbonate, and acetylation, the concentration of cAMP and cGMP in the extract was measured using the ^125^I radioimmunoassay kit according to manufacturer's instructions (Amersham Pharmacia Biotech).

### RBD binding assay

The RBD binding assay was performed as described elsewhere [30]. Briefly, 6 h or 10 h pulsed cells were washed twice and resuspended at 5 × 10^7 ^cell/ml in Soerensen phosphate buffer. Cells were stimulated with 200 nM cAMP, aliquots (0.5 ml) were selected at the indicated time points and lysed in an equal volume of ice-cold 2× HK-LB (20 mM sodium phosphate pH 7.2, 2% Triton X-100, 20% glycerol, 300 mM NaCl, 20 mM MgCl_2_, 2 mM EDTA, 2 mM Na_3_VO_4_, 10 mM NaF, with protease inhibitor Roche), and incubated on ice for 5 min. The lysates were cleared by centrifugation for 10 min and protein concentrations were determined using DC Protein Assay (Bio-Rad). A 0.8 mg portion of protein lysates was incubated with 100 μg of GST-Byr2 (RBD) and the mixture was incubated at 4°C for 1 h. Beads were harvested by centrifugation and washed three times in 1× HK-LB. A volume of 40 μl of 1× SDS gel loading buffer was added to the pellet beads and the mixture was boiled for 5 min. Samples were fractionated by SDS-PAGE, blotted, blocked and probed with RasC or RasG antibody. Bands were determined by enhanced chemiluminescence reaction (Amersham Pharmacia Biotech).

### Molecular cloning and sequence analysis

The *rasGEFM *gene was isolated using PCR based method. Through the sequence derived from DNA database screening [13,14] a pair of primers was designed (5'-ATGATGAATGAAGTTTCTTCAAATTC-3' and 5'-CCATCGATAATTATCTAAATAATGGATTTGA-3') and used for PCR amplification. As template, cDNA isolated with "First strand cDNA synthesis kit" (Amersham Pharmacia Biotech), from RNA of cells developed for 5 hours on solid substrata, was used. The DNA fragment, of approximately 2.7 kb, was then ligated into "pGEM-T easy vector" (PROMEGA) and cloned into DH5α *E. coli *strain. PCR products were purified from gel with "High Pure PCR Product Purification" kit (Roche) and verified by sequencing.

### Construction of the *D.d. rasGEFM *null strain (HSB61)

To construct the *rasGEFM *null strain the *rasGEFM *locus was disrupted via homologous recombination. The blasticidin resistance gene (*bsr*), used as selectable marker, was excised from pUCBsrΔ Bam [60] with *Hind*III and *Xba*I and subsequently inserted into the JC2a86b07.s1 clone (*Dictyostelium *genome project) digested with *Hind*III and *Xba*I. Subsequently the N-terminal portion (from position 0 to 617 bp) of the gene was ligated into *Kpn*I site of the above vector, using the "DNA ligation kit" (Amersham Pharmacia Biotech). The vector, carrying the selectable marker was electroporated [61] into parental strain and transformed cells selected for blasticidin resistence. Resistant cells were cloned, and clones subsequently screened via Southern blot in order to identify *Dictyostelium *clones in which the *RasGEF M *locus was disrupted.

### Southern and Northern hybridization analysis

Genomic DNA was extracted and purified by CsCl gradient centrifugation as previously described by Nellen, *et al*. [62], digested with *EcoR*I, run onto 0.8% agarose gel, blotted onto Hybond-N membrane (Amersham Pharmacia Biotech), and subjected to Southern assay [63]. The membrane was probed with a 800 bp *RasGEF M *cDNA specific probe, corresponding to bp. 760–1518 of the cDNA clone, previously radiolabelled by the "Megaprimer™DNA Labelling System" using [α^32^] dATP (Amersham).

For Northern blots, total RNA was prepared using TRIZOL reagent (GIBCO) according to manifacturer's instructions. RNA was then resuspended in DEPC treated water, quantified, and 15 μg were size separated on 1.2% agarose gel in presence of formaldehyde. Equal loading of samples, was checked by probing membranes with the *actin *gene. The radiolabelled DNA fragments used as probes were as follow: *car1*, *csA*, *acaA *(fragment from 2.7 kb to 4.2 kb of cDNA clone), *RasGEF M*. After being hybridised with the first cDNA probe, Northern blots were stripped with 0.1% SDS in boiling water and then re-hybridised with a second probe.

## Authors' contributions

M. A. and E. B. carried most of the experiments and were involved in drafting the manuscript. D. L. was responsible for light scattering oscillations and Ca^2+ ^analysis, H. K. and G. W. for pull down experiments with RasC and RasG. S. B. supervised the work and revised the manuscript, with contributions by all authors.

## Supplementary Material

Additional File 1 **Chemotaxis of AX2 after 5 hours of starvation.mov: 6.5 MB**. Cells were seeded onto 35 mm/glass base dish (Iwaki) and subjected to a cAMP filled micropipette assay, as described in Material and Methods. Images were recorded in a Panasonic videorecorder (AG-TL700) with a ZVS-47 DE camera (Zeiss) mounted on an Axiovert HAL100, using Neofluar 100X/1.3 oil immersion objective and DIC filter. The recorded time-lapse movie was transferred to a computer using USB Istant Video (ADS Technologies). Click here for file

Additional File 2 Chemotaxis of HSB61 after 5 hours of starvation.mov: 6.9 MB Click here for file

Additional File 3 Chemotaxis of HSB61 starved and pulsed with cAMP for 10 hours.mov: 9.9 MB Click here for file
